# Neobavaisoflavone ameliorates LPS-induced RAW264.7 cell inflammations by suppressing the activation of NF-κB and MAPKs signaling pathways 

**DOI:** 10.22038/IJBMS.2022.65372.14389

**Published:** 2022-08

**Authors:** Qing Yuan, Jing Wang, Lichen Guo, Yao Xu, Limin Hu, Haoping Mao, Lin Miao, Han Zhang, Lijuan Chai

**Affiliations:** 1 State Key Laboratory of Component-based Chinese Medicine, Key Laboratory of Pharmacology of Traditional Chinese Medical Formulae, Ministry of Education, Tianjin University of Traditional Chinese Medicine, Tianjin, China; 2 Tianjin Key Laboratory of Traditional Chinese medicine Pharmacology, Tianjin University of Traditional Chinese Medicine, Tianjin, China; 3 China Resources Sanjiu Medical & Pharmaceutical Co., Ltd, Shenzhen, China; # These authors contributed eqully to this work

**Keywords:** Inflammation, MAPK signaling system, Neobavaisoflavone, NF-kappa B

## Abstract

**Objective(s):**

Neobavaisoflavone (NBIF) is an isoflavone isolated from *Psoralea corylifolia* L. It can effectively regulate the redox state as a natural anti-oxidant and show some anti-inflammatory activity. However, its molecular mechanism is poorly studied. In this study, RAW264.7 cells were treated with lipopolysaccharide (LPS) to investigate the anti-inflammatory activity and potential NBIF mechanism.

**Materials and Methods:**

RAW264.7 cells were treated with LPS (62.5 ng/ml) and exposed to different concentrations of NBIF (0.01, 0.1, and 1 μM) for 24 hr. Inflammatory cytokines of RAW264.7 cells were measured by the Griess method, ELISA, and western blot. Phagocytosis of RAW264.7 macrophages was measured by FITC-dextran uptake assay. The phosphorylation protein expression levels of MAPKs (JNK, p38, and ERK), NF-κB p65, IκBα, and IκB kinase were analyzed by western blot. The results were analyzed using one-way ANOVA with Tukey’s multiple comparison test.

**Results:**

NBIF significantly inhibited NO and ROS production by down-regulation of iNOS and COX-2 protein expression. Additionally, the amount of release and protein levels of inflammation cytokines IL-6, IL-1β, and TNF-α were significantly decreased by NBIF. Moreover, FITC–dextran uptake assay by flow cytometry presented that NBIF significantly enhanced the phagocytic capacity of RAW264.7. Mechanistically, NBIF significantly down-regulated MAPK activation and inhibited the nuclear translocation of NF-κB p65.

**Conclusion:**

The present study demonstrates that NBIF inhibited inflammation and enhanced the phagocytic capacity of RAW264.7 cell-related MAPKs and NF-κB signaling pathways induced by LPS. These findings suggest that NBIF may have clinical utility as an anti-inflammatory agent.

## Introduction

Inflammation, as an organism’s defensive response, involves various cells and cytokines; moderate inflammation can defend the organism from exogenous stimuli (1). However, persistent inflammation may lead to various chronic inflammatory diseases, such as atherosclerosis and osteoporosis (2). Macrophages are crucial immune cells in the body and regulate inflammatory mediator production when they are exposed to a dose of LPS (3). The release of inflammatory mediators such as NO, TNF-α, IL-6, and IL-1β by macrophages leads to an imbalance in the micro-environment of adjacent tissues (4-5). NO-induced overexpression by iNOS and COX-2 in macrophages plays a key role in developing inflammatory diseases (6). Simultaneously, phagocytosis of damaged cells by macrophages is an important part of the inflammation treatment and plays an important role in maintaining body homeostasis (7). Therefore, reducing inflammatory factor expression and promoting phagocytosis by macrophages are therapeutic strategies for many inflammatory diseases.

Mouse mononuclear macrophage leukemia cells RAW264.7 are often used in laboratories to simulate *in vitro* inflammatory responses due to their strong adhesion and ability to phagocytose antigens (8). LPS, a component of the cell wall of Gram-negative bacteria, can stimulate a significant inflammatory response in the organism (9). When the body is exposed to high doses of LPS, macrophages can be activated to secrete inflammatory mediators such as TNF-α, IL-1β, and IL-6, thereby inducing inflammatory responses.

MAPK and NF-κB signaling pathways are closely related to immune and inflammatory responses (10). NF-κB and MAPK activation initiate the transcription and inflammatory gene expression, accumulating various mediators, such as iNOS, COX-2, TNF-α, IL-6, and IL-1β (11, 12). It is an important step in the inflammation treatment by inhibiting excessive production of inflammatory cytokines and mediators via NF-κB and MAPKs pathways; also, it is an important target for drug development and discovery. 

*Psoralea corylifolia *L. (PLC) has been extensively used as a traditional Chinese medicine for centuries to prevent and treat many diseases, such as osteoporosis, anthelmintic, diuretic, vitiligo, and specific skin diseases (13). Recent studies have revealed that PLC active components of seeds and roots, mainly bakuchiol, bavachin, corylin, neobavaisoflavone (NBIF), and isobavachalcone, exhibit antimicrobial, anti-oxidant, and immunomodulatory properties (14). A previous study reported that NBIF inhibits NO production and cytokines IL-1*β*, IL-6, and TNF-*α* in INF-γ plus LPS-induced RAW264.7 macrophages, showing certain anti-inflammatory activity (15). Hence, this bioactive compound holds promise to cure inflammatory diseases. However, reduced NO production by NBIF and its anti-inflammatory mechanism are poorly studied. In this study, NBIF’s effect on the expression of the inflammatory factors and its possible mechanism was detected by LPS stimulation of RAW264.7 to establish an inflammatory model. This study aims to investigate the anti-inflammatory effect, NBIF mechanism, and its possible clinical application.

## Materials and Methods

***Materials and reagents***

**Figure 1:**
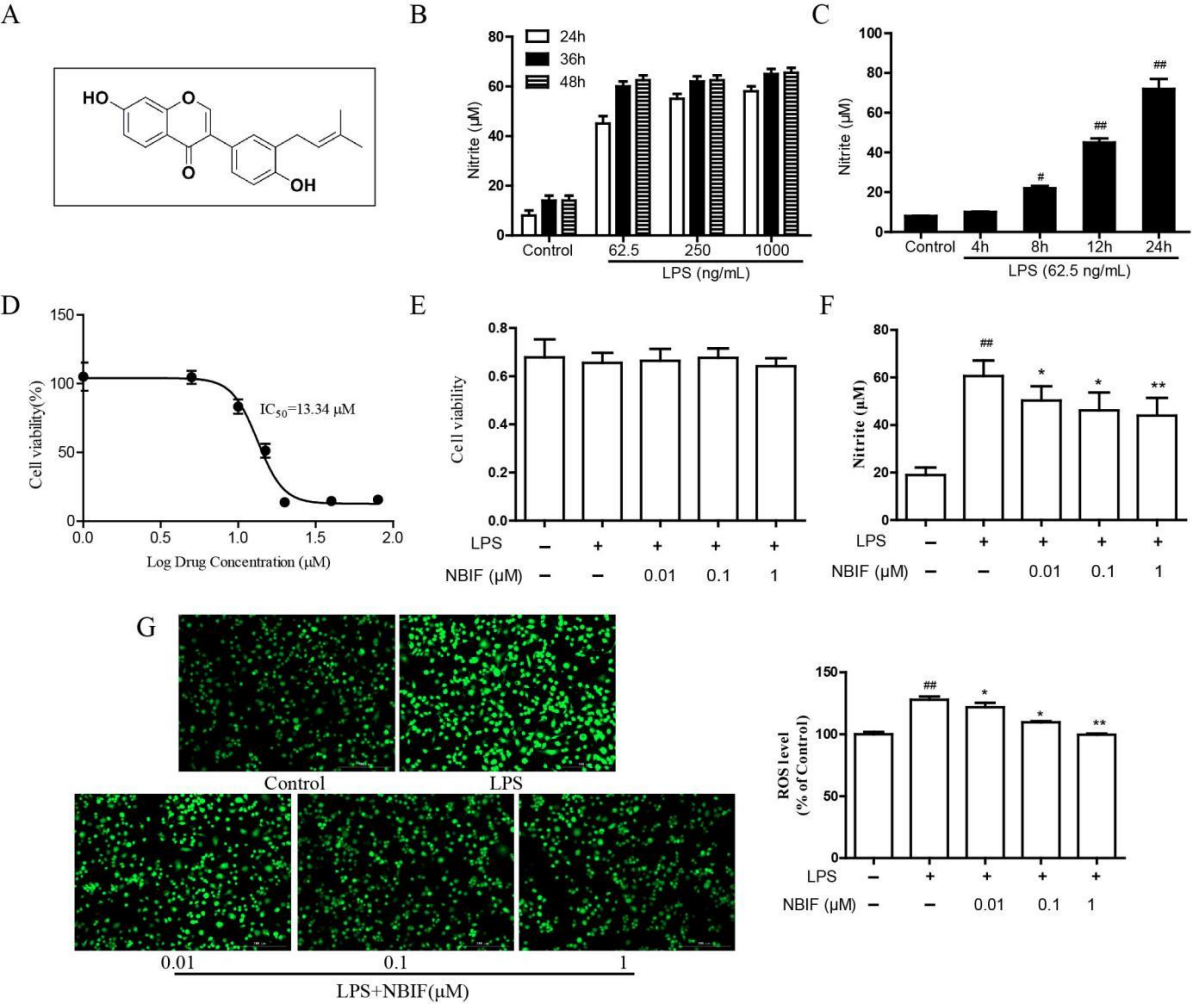
Model establishment and effects of NBIF on cell viability, NO production, and ROS expression in LPS-induced RAW264.7 cells. (A) Chemical construction of NBIF. (B) NO production of RAW264.7 cells in various concentrations (62.5, 250, and 1000 ng/ml) of LPS for 24, 36, and 48 hr. (C) NO production of RAW264.7 cells for different times (4, 8, 12, and 24 hr) was treated with 62.5 ng/ml LPS. (D) IC_50 _values of NBIF in RAW264.7 cells. (E) Effects of NBIF (0.01, 0.1, and 1 μM) on cell viability in RAW264.7 cells stimulated with LPS (62.5 ng/ml) for 24 hr. (F) Effects of NBIF (0.01, 0.1, and 1 μM) on NO production in RAW264.7 cells stimulated with LPS (62.5 ng/ml) for 24 hr. (G) Intracellular ROS levels were detected by fluorescence analysis. Representative images of ROS (green): they were Control group, LPS-induced group, NBIF (0.01 μM), NBIF (0. 1 μM), and NBIF (1 μM) group, respectively. Scale bars: 200 μm. The data are expressed as mean ± SD, n=6. # *P*<0.05 vs Control, * *P*<0.05 vs LPS-induced group

NBIF: Neobavaisoflavone; ROS: Reactive oxygen species

LPS(#L2880), FITC–dextran (#FD40S), and DMSO (#D2650), were purchased from Sigma (St. Louis, MO, USA). Fetal bovine serum (FBS, #10099-141) and Dulbecco’s modified Eagle medium (DMEM, #C11995500BT) were purchased from Invitrogen Gibco (Grand Island, NY). The mouse TNF-α (#MTA0008), IL-6 (#M6000B), and IL-1β (#MLB00C) ELISA kits were acquired from R&D (USA). Antibodies against NF-κB (#3032), p-IKK (#2697), IKK (#2697), p-IκBα (#2859), IκBα (#4812), p-ERK (#9101), ERK (#9102), p-p38 (#9211), p38 (#9212), JNK (#9252), p-JNK (#4668), and β-actin (#4967) were obtained from Cell Signaling Technology (Beverly, MA, USA). Antibodies against TNF-α (#ab183218), IL-6 (#ab259341), IL-1β (#ab254360), iNOS (#ab178945), COX-2 (#ab179800), LaminB (#ab171123) were obtained from Abcam (Cambridge, MA, USA). The nuclear protein extraction kit (#R0050) was purchased from Beyotime (Jiangsu, China). Additional chemical reagents were purchased from Tianjin University (Tianjin, China). NBIF was purchased from National Institutes for Food and Drug Control, Purity (HPLC) ≥ 98% (Beijing, China) and dissolved in DMSO. The chemical construction of NBIF was shown in Figure 1A.

***Cell culture and treatment***

RAW264.7 cells were obtained from the Chinese Academy of Medical Sciences, Institute of Cell Resource Center, and used for less than 20 passages. We cultured RAW264.7 in a DMEM medium containing 10% FBS, 100 U/L penicillin, and 100 mg/L streptomycins at 37 °C under 5% CO_2 _humidified conditions. The culture medium was exchanged every three days.

***Nitrite assay***

The supernatant NO production in RAW264.7 cells was analyzed by the Griess reagent. Briefly, culture supernatant (100 μl) in each well was incubated for 10 min at room temperature with 200 μl of Griess reagent. The absorbance was measured at 540 nm using a TECAN micro-plate reader (INFINITE F50, TECAN, Switzerland). 

***Cell viability assay***

RAW264.7 cells were incubated with different concentrations of NBIF (0.01, 0.1, and 1 μM) for 24 hr. Subsequently, 100 μl MTT (#M5655, 0.5 mg/ml) was added, and incubated for 4 hr at 37 °C. Then, the culture supernatant was discarded, and the cells were shaken for 10 min with 200 μl DMSO. The absorbance was measured at 490 nm using a TECAN micro-plate reader (INFINITE F50, TECAN, Switzerland). 

***Detection of intracellular reactive oxygen species (ROS)***

LPS-treated RAW264.7 cells were incubated with different concentrations of NBIF (0.01, 0.1, and 1 μM) for 24 hr. Then, 5 μM CM-H_2_DCFDA (#AM1830) was added to the plates for 10 min at 37 °C to determine the ROS level. The ex/em wavelengths of 488/525 nm were measured under a fluorescent microplate reader (Spark, TECAN, Switzerland). Images were captured under a ﬂuorescence microscope (Leica Microsystems, GER).

***Enzyme-linked immunosorbent assay (ELISA)***

LPS-treated RAW264.7 cells were incubated with different concentrations of NBIF (0.01, 0.1, and 1 μM) for 24 hr. Then, the cell culture supernatants were collected, and ELISA kits were used to detect the secretion of TNF-α, IL-6, and IL-1β as described by the manufacturer.

***Phagocytosis assay***

Following treatment of RAW264.7 cells as above, they were centrifuged and resuspended with PBS (100 μl) containing 2% FBS, and 5 μl of FITC-dextran was added to the cell suspension. Cells were washed with cold PBS after 30 min of incubation. After centrifugation, the cells were resuspended with 500 μl PBS, filtered through a membrane, and detected by flow cytometry (FACS Calibur, BD) for phagocytosis and fluorescence intensity. Images of RAW264.7 cell phagocytosis of dextran were captured using an inverted fluorescence microscope (Spark, TECAN, Switzerland).

***Western blot***

After washing three times with cold PBS, RAW264.7 cells were lysed in RIPA solution containing 1 mM PMSF for 20 min. Protein concentration was measured by the Bradford assay. After mixing cell extracts (20 μg) with 5 × SDS sample buffer for 10 min at 95 °C, electrophoresis was performed on a 10% SDS gel and then transferred to PVDF membranes. After 2 hr of incubation with 5% nonfat milk, the membranes were incubated with the first antibody (iNOS, COX-2, TNF-α, IL-6, IL-1β, NF-κB/p65, p-IKK, IKK, p-IκBα, IκBα, p-ERK, ERK, p-p38, p38, JNK, p-JNK, Laminin-B, and β-actin) at 4 °C overnight with gentle shaking. After four washes with TBST, the membranes were incubated for 1 hr at room temperature with a second HRP-conjugated antibody. Blotting bands were detected with ECL reagents. Bands were quantified based on optical density values using the ChemDoc MP system (Bio-Rad, Hercules, CA, USA). 

***Statistics***

The data were expressed as mean ± SD. SPSS software version 18.0 was used for statistical analysis. The results were analyzed using one-way ANOVA with Tukey’s multiple comparison test. *P*-values of less than 0.05 were considered statistically significant (*P*<0.05).

## Results

***Model establishment***

We studied the LPS concentration and its action time to determine the best model conditions. The induction of various concentrations (62.5, 250, and 1000 ng/ml) of LPS at 24–48 hr significantly increased the NO production, but there was no significant difference between different LPS concentrations (Figure 1B). A further study verified the effect of 62.5 ng/ml LPS stimulation on NO expression within 24 hr. NO expression increased significantly at 8 hr, and its expression continued to increase at 24 hr (Figure 1C). Therefore, 62.5 ng/ml LPS stimulating RAW264.7 for 24 hr was employed hereafter. 

***Effects of NBIF on cell viability in LPS-induced RAW264.7***

Figure 1D displays that the IC_50_ value of NBIF is 13.34 uM. Considering that 10 μM NBIF has a certain inhibitory effect on cell viability (83.35%), we selected 0.01, 0.1, and 1 μM concentrations of NBIF in the following anti-inflammatory experiments. We cultured RAW264.7 cells with NBIF at concentrations of 0.01, 0.1, and 1 μM for 24 hr to investigate the NBIF cytotoxicity. Figure 1E shows no significant decrease in cell survival for NBIF concentrations at 0.01, 0.1, and 1 μM.

***Effect of NBIF on NO production in LPS-induced RAW264.7***

NO production was examined in RAW264.7 exposed to inflammatory stimuli to investigate the NBIF anti-inflammation activity. Figure 1F displays that NO production was significantly up-regulated in LPS-induced group cells, which was dose-dependent and reduced by NBIF at concentrations of 0.01, 0.1, and 1 μM. 

***Effects of NBIF on ROS expression in LPS-induced RAW264.7***

ROS is also one of the signals that mediate inflammatory responses. ROS production in cells was increased in the LPS-induced group compared with the control group. NBIF (0.01, 0.1, and 1 μM) significantly reduced ROS levels in a concentration-dependent manner compared with the LPS- induced group (Figure 1G). 

***Effects of NBIF on pro-inflammatory cytokine expression in LPS-induced RAW264.7***

**Figure 2:**
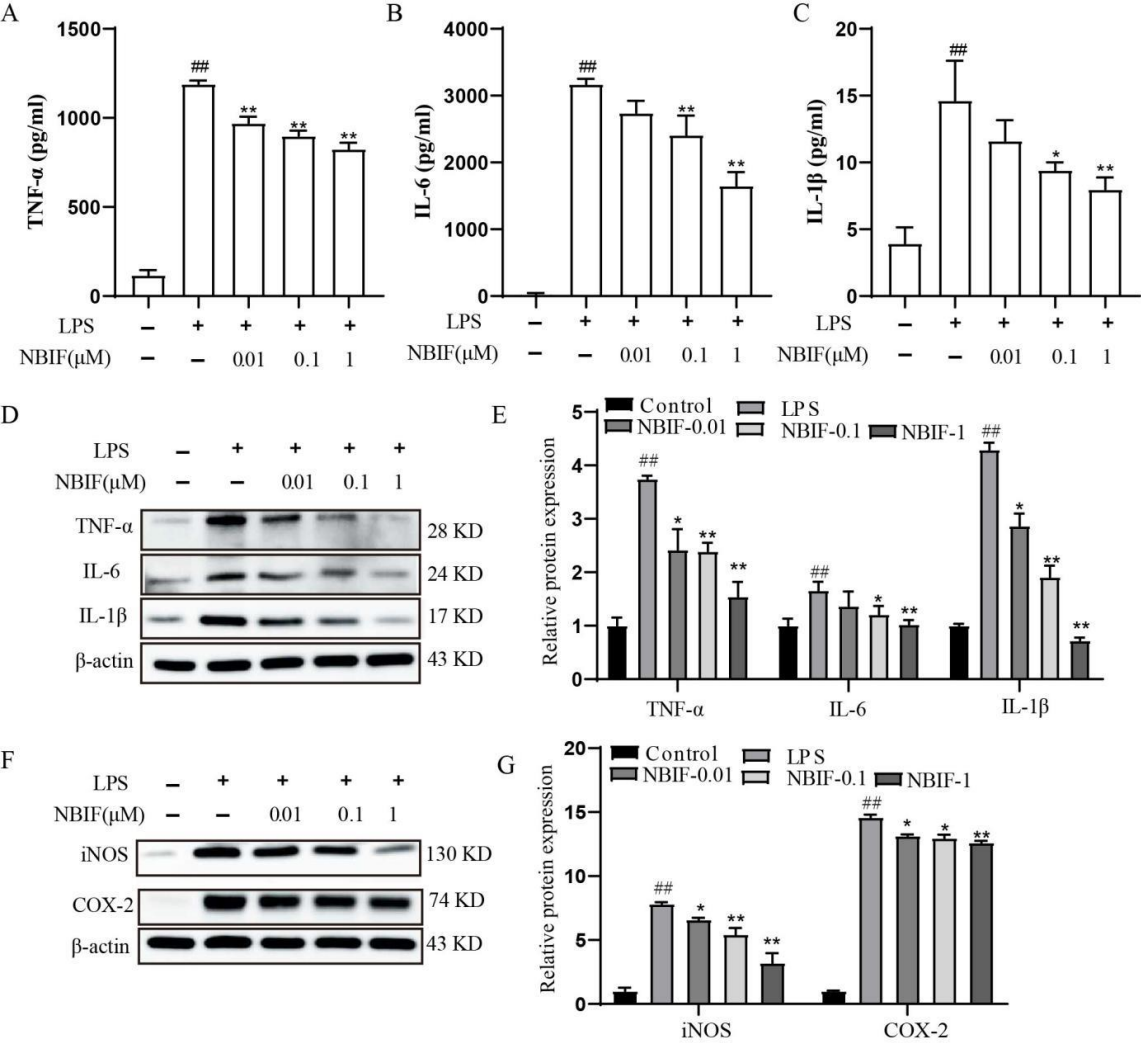
Effect of NBIF on the expression of inflammatory factors and iNOS/COX-2 proteins in LPS-inducedRAW264.7 cells. (A-C) Release in the culture medium of TNF-α, IL-6, and IL-1β was detected by ELISA assay. (D-F) Protein expressions of TNF-α, IL-6, and IL-1β were detected by western blot assay. (E-G) iNOS and COX-2 protein expression levels were detected by western blot assay. Relative protein expression was expressed as optical density value relative to the control group after normalizing to β-actin optical density value. The data are expressed as mean ± SD, n=3. # *P*<0.05 vs Control, * *P*<0.05 vs LPS-induced group

NBIF: Neobavaisoflavone; ROS: Reactive oxygen species

We further evaluated the anti-inflammatory effect of NBIF on LPS-stimulated inflammatory cytokines by examining the secretion of TNF-α, IL-6, and IL-1β by RAW264.7 cells after LPS induction. The ELISA and Western blot experiments showed that the secretion and protein expressions of TNF-α, IL-6, and IL-1β were significantly up-regulated in the LPS-induced group compared with the control group and significantly decreased by treatment with NBIF (0.01, 0.1, and 1 **μM**; Figure 2A-D). 

***Effect of NBIF on iNOS and COX-2 protein expression in LPS-induced RAW264.7***

NO production induced by iNOS (an enzyme that induces NO synthesis) is regulated via COX2 expression. We investigated iNOS generation and COX-2 expression to study the inhibitory mechanism of NO production. Figure 2E-F demonstrates that the protein expressions of iNOS and COX-2 were significantly up-regulated in the LPS-induced group compared with the control group. The iNOS and COX-2 protein levels were decreased in a concentration-dependent manner after being treated by NBIF at concentrations of 0.01, 0.1, and 1 μM compared with the LPS-induced group.

***Effect of NBIF on phagocytosis in LPS-induced RAW264.7***

**Figure 3:**
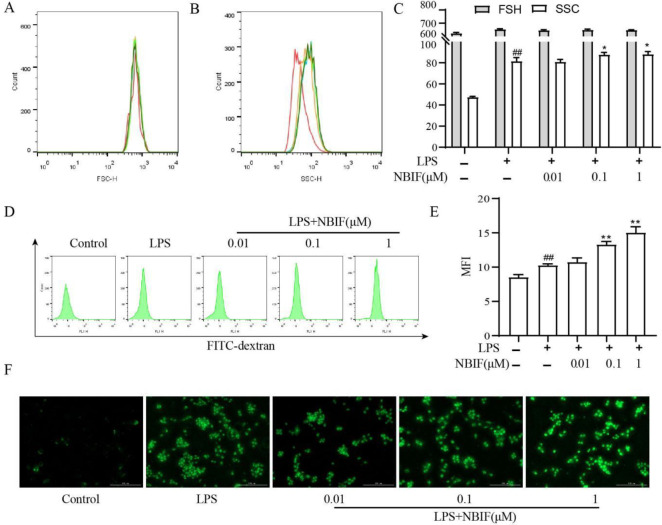
Effect of NBIF on phagocytosis in LPS-induced RAW264.7 cells. (A-C) Cell size and intracellular complexity were determined by detecting RAW264.7 cells’ FSC and SSC signals by flow cytometry. (D-E) Effect of NBIF (0.01, 0.1, and 1 μM) on phagocytosis of RAW264.7 cells was detected by flow cytometry, internalization of FITC-dextran was used as an evaluation index. Intracellular FITC-dextran fluorescence intensity was expressed as Median Fluorescence Intensity (MFI). (F) Representative images of uptake of FITC-dextran (green): they were Control group, LPS-induced group, NBIF (0.01 μM), NBIF (0. 1 μM), NBIF (1 μM) group, respectively. Scale bars: 200 μm. Data are expressed as mean ± SD, n=3. # *P*<0.05 vs Control, * *P*<0.05 vs LPS-induced group

NBIF: Neobavaisoflavone; ROS: Reactive oxygen speciesc

As macrophage phagocytosis is an integral part of regulation of the inflammatory response, we investigated the NBIF effect on macrophage phagocytosis in LPS-induced RAW264.7. Side scatter (SSC) and forward scatter (FSC) reflected intracellular complexity and object size, respectively. Figure 3A-C displays that LPS-induced SSC signals in the RAW264.7 cell group were significantly enhanced compared with the control group. The uptake of FITC-dextran by RAW264.7 cells increased with higher NBIF concentrations (0.1 and 1 μM) after LPS stimulation compared with the LPS-induced group (Figure 3D-E). These results suggest that NBIF significantly enhanced the LPS-induced phagocytosis in RAW264.7 cells.

***Effect of NBIF on MAP kinase activation in LPS-induced RAW264.7***

**Figure 4:**
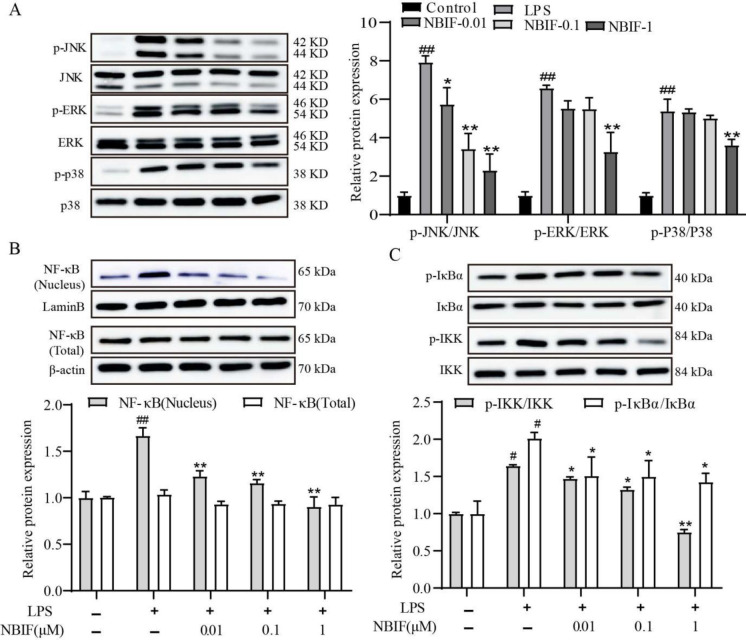
Effect of NBIF on nuclear translocation of NF-κB and MAPKs phosphorylation in LPS-induced RAW264.7 cells. (A) Protein expressions of p-ERK, ERK, p-JNK, JNK, p-p38, and p38 were detected by Western blotting assay. (B) Protein levels of nuclear NF-κB, cytoplasmic NF-κB, whole cell phosphorylated IκBα, and phosphorylated IKK were detected by western blot. The data are expressed as mean ± SD, n=3. # *P*<0.05 vs Control. * *P*<0.05 vs LPS-induced group

NBIF: Neobavaisoflavone; ROS: Reactive oxygen species

We investigated the phosphorylation of ERK1/2, p38, and JNK for RAW264.7 inflammation by Western blot to address a potential role for MAPK during these processes. The results indicate that activation of RAW264.7 inflammatory response by LPS leads to phosphorylation of ERK1/2, p38, and JNK. Additionally, JNK phosphorylation was significantly suppressed by NBIF at concentrations of 0.01, 0.1, and 1 μM compared with the LPS-induced group. ERK1/2 and p38 phosphorylation was significantly suppressed by NBIF at 1 μM compared with the LPS-induced group (Figure 4A).

***Effect of NBIF on nuclear translocation of NF-*** ***κB*** ***in LPS-induced RAW264.7***

The results revealed that the nuclear NF-κB/p65 levels in the nucleus were significantly increased in the LPS-induced group compared with the control group and significantly reduced by administration of NBIF (0.01, 0.1, and 1 μM). NF-κB/p65 total protein levels did not vary significantly between groups (Figure 4B). Meanwhile, phosphorylation of IκBα and IKK protein levels in the cytoplasm was significantly increased in the LPS-induced group compared with the control group and were all substantially reduced by administration of NBIF (0.01, 0.1, and 1 μM) (Figure 4C). The results suggest that NBIF inhibited activation of the NF-κB pathway by inhibiting p65 subunit translocation into the nucleus.

**Figure 5:**
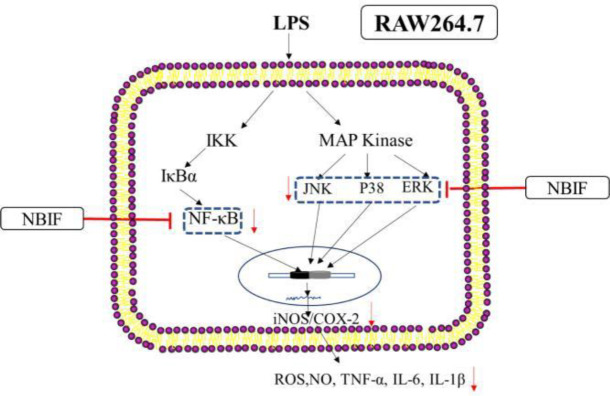
Possible mechanisms of NBIF against LPS-induced inflammation in RAW264.7 cells

NBIF: Neobavaisoflavone; LPS: Lipopolysaccharide

## Discussion

NBIF is an isoflavone mainly isolated from *P. corylifolia L* that has shown anti-oxidant, anti-bacterial, and anti-tumor activities (16). Considering that inflammation plays a pivotal role in osteoporosis (17), studies have indicated that NBIF could enhance osteoblastic cell differentiation in osteoblastic MC3T3-E1 cells (18-19), suggesting that NBIF may be a useful candidate for treating inflammatory diseases. In this study, we aimed to investigate the anti-inflammatory activity and molecular mechanism of NBIF.

LPS is an effective macrophage inducer and a key mediator of natural immune response (20). LPS stimulation of RAW264.7 macrophages results in intracellular signal transmission, producing pro-inflammatory cytokines such as NO and other pro-inflammatory mediators (21-22). Additionally, the pro-inflammatory cytokines TNF-α, IL-6, and IL-1β contribute to inflammatory diseases (23). iNOS is the rate-limiting enzyme for NO synthesis and mainly exerts biological effects on the body. Similarly, the inflammatory mediator NO can promote the release of inflammatory cytokine COX-2, a key enzyme for inflammatory cells to aggravate the inflammatory response. Therefore, regulation of iNOS/COX-2-mediated release of inflammatory factors is an important target for treating inflammatory responses (24). To verify whether NBIF has anti-inflammatory activity, we studied its inhibitory effects on NO production and expression of iNOS, COX-2, and proinflammatory cytokines TNF-α, IL-6, and IL-1β in the RAW264.7 induced by LPS. The results showed that NBIF inhibited NO production by reducing the LPS-induced expression of iNOS and COX-2. Moreover, NBIF reduced LPS-induced TNF-α, IL-6, IL-1β production. The results indicated that NBIF has certain anti-inflammatory activity on RAW264.7 cells induced by LPS. 

Phagocytic capacity is an important indicator in detecting macrophage function. As an important cell regulating immune response, LPS stimulates macrophages to secrete large amounts of NO, ROS, pro-inflammatory cytokines, and phagocytose-damaged apoptotic cells (25). Flow cytometry was used to detect the uptake of FITC-dextran by RAW264.7 to determine the phagocytic capacity of RAW264.7 cells. The intensity of the forward FSC can reflect the size of cells, while the intensity of the side SSC is related to the properties of particles in cells, which can judge the complexity of cells. The phagocytosis results by flow cytometry showed no significant change in FSC value in each group, indicating that the cell size did not change. While SSC value of NBIF changed significantly, suggesting that the increase of FITC-dextran phagocytosis by RAW264.7 may lead to the changes in intracellular complexity. Additionally, inverted fluorescence microscopy showed that the uptake of FITC-dextran by NBIF was significantly enhanced. Our results speculate that NBIF may alleviate the inflammatory response by enhancing the phagocytosis of LPS-induced macrophages.

According to previous research, NF-κB and MAPK are essential signaling pathways for LPS-activated inflammation (26). The inhibitory factor IκBα specifically localizes NF-κB in the cytoplasm during the resting state. IκBα is phosphorylated, and NF-κB is separated from the IκBα complex in response to LPS stimulation, resulting in the continuous activation of NF-κB. Furthermore, the cytoplasm transfers to the nucleus, binds to the target gene promoter, and activates transcription, which leads to the synthesis and secretion of the inflammatory mediator NO and cytokines TNF-α, IL-6, and IL-1β (27, 28). Therefore, it can be used as the target of anti-inflammatory drugs by inhibiting the NF-κB signal transduction pathway. The results showed that LPS could significantly up-regulate the phosphorylation level of IκBα, while NBIF could significantly inhibit the phosphorylation of IKK, IκBα, and p65 NF-κB activation in LPS-induced RAW264.7 macrophages in a dose-dependent manner. These results indicated that NBIF inhibited NO and pro-inflammatory cytokine levels by suppressing the NF-κB pathway to exert its anti-inflammatory effect.

It has been demonstrated that MAPKs regulate pro-inflammatory cytokine production (29). Various extracellular molecular signals can activate the MAPKs signaling pathway, its activation induces phosphorylation of downstream signaling molecules and is involved in processes such as cell proliferation, inflammation, and apoptosis (30). MAPKs regulate the expression of pro-inflammatory mediators and are a major component of inflammation-associated signaling pathways. Additionally, p38 is involved in the transcriptional regulation of pro-inflammatory mediators, including iNOS, COX-2, and TNF-α, during LPS-induced macrophage responses (31). Among LPS-induced macrophage responses, ERK phosphorylation is believed to be associated with increased pro-inflammatory cytokine and iNOS expression (32). Several studies reported significant role of JNK during LPS-stimulated macrophage expression of iNOS and COX-2 (33). Consequently, the MAPK signaling pathway was evaluated to illustrate the anti-inflammatory mechanism of NBIF in LPS-induced macrophages. Consistent with earlier findings, our results showed that NBIF inhibited the phosphorylation of p38 MAPKs, JNK, and ERK induced by LPS.

The present study showed that LPS induced NO and ROS production and expression of TNF-α, IL-6, and IL-1β in RAW264.7 cells, which NBIF could inhibit. Furthermore, reduced NO production and TNF-α, IL-6, and IL-1β were due to inhibiting iNOS and COX-2, key enzymes for NO production under inflammatory conditions. Moreover, MAPKs and NF-κB activity in LPS-induced RAW264.7 were significantly alleviated by NBIF, indicating the potential application prospects in treating inflammatory diseases. 

## Conclusion

Our findings suggest that the anti-inflammatory effect of NBIF extracted from PLC is exerted by inhibiting LPS-stimulated expression of NO, ROS, iNOS, and COX-2 inflammation-related TNF-α, IL-6, and IL-1β, inhibiting transcription factor NF-κB and  MAPKs pathway, as well as enhancing macrophage phagocytosis (the proposed mechanism of NBIF is depicted in Figure 5). Our results indicate that NBIF can potentially treat inflammatory diseases as an anti-inflammatory agent.

## Authors’ Contributions

YQ, CLJ, WJ, and ZH Designed the experiments; YQ, CLJ, and XY Performed experiments and collected data; YQ, WJ, CLJ, and XY Discussed the results and strategy; GLC, MHP, and ML Prepared the draft manuscript; CLJ, ZH, and HLM Edited the article; ZH and HLM Supervised, Directed and managed the study; YQ, WJ, GLC, XY, HLM, MHP, ML, ZH, and CLJ Approved the final version to be published.

## Funding

 This research was funded by the National Natural Science Foundation of China (No.81102860, No.81573644, No.81622051). 

## Conflicts of Interest

The authors declare no conflict of interest. 
